# Cancer history and other personal factors affect quality of life in patients with hepatitis C

**DOI:** 10.1186/1477-7525-3-39

**Published:** 2005-06-16

**Authors:** Sara H Olson, Sandy Iyer, Jennifer Scott, Orry Erez, Shelby Samuel, Temima Markovits, Myron Schwartz, Charlene Toro, Maya Gambarin-Gelwan, Robert C Kurtz

**Affiliations:** 1 Unversity of Michigan Medical School, 1301 Catherine Road, Ann Arbor, MI 48109, USA; 2 Department of Medicine, North General Hospital, 50 East 118^th ^St., New York, NY 10035, USA; 3 Department of Surgery, Mount Sinai School of Medicine, 1 Gustave L. Levy Place, 1190 5^th ^Ave., New York, NY 10029, USA; 4 Department of Medicine, Mount Sinai School of Medicine, 1 Gustave L. Levy Place, 1190 5^th ^Ave., New York, NY 10029, USA; 5 Department of Medicine, Memorial Sloan-Kettering Cancer Center, 1275 York Ave., New York, NY 10021, USA; 6 Bronx Veteran Affairs Medical Center, Bronx, NY 10468, USA

## Abstract

**Background:**

Although patients with chronic hepatitis C (CHC) have been found to have reduced quality of life, little is known about how other characteristics affect their quality of life. The purpose of this study was to investigate the effect of other characteristics, including history of cancer, on quality of life in patients with CHC.

**Methods:**

One hundred forty patients from clinics at three hospitals in New York City completed a detailed epidemiologic interview about demographic and lifestyle characteristics and the SF-36 measuring health-related quality of life. We compared results from our patients to normative data using t-tests of differences between means. We used multivariate analyses to determine other personal and health-related factors associated with quality of life outcomes.

**Results:**

Compared to normative data, these patients had reduced quality of life, particularly on physical functioning. The summary Physical Component Score (PCS) was 45.4 ± 10.6 and the Mental Component Score (MCS) was 48.2 ± 11.1, vs norms of 50 ± 10.0; p-values were <0.0001 and <0.05, respectively. In multivariate analyses, the PCS was significantly lower among those with cancer history, ≥ 2 other chronic conditions, less education, low physical activity, and higher alanine aminotransferase (ALT) levels. Cancer was more important for men, while other chronic conditions were more important for women. On the MCS, history of depression, low physical activity, alcohol use, and female gender were independently associated with poorer scores.

**Conclusion:**

Several health and lifestyle factors independently influence quality of life in CHC patients. Different factors are important for men and women.

## Background

Several studies investigating health-related quality of life have found reduced quality of life in patients with chronic hepatitis C (CHC), particularly on measures relating to physical functioning. Because of the types of exposures leading to CHC, these patients are likely to have other demographic, lifestyle, and health-related factors that affect their quality of life; such factors have not been well-evaluated in previous studies. Most studies of quality of life in this patient population have been conducted among patients who were taking part in clinical trials [1-6]. There has been less emphasis on the quality of life of patients in a clinic setting, who are more typical of patients with CHC. Our study of hepatitis C in three hospitals in New York City includes patients from a cancer center, many of whom have had cancer, and patients from a community hospital in Harlem.

We collected information on quality of life and detailed information on other lifestyle and health factors that are likely to be related to quality of life. We hypothesized that health-related quality of life among these patients would be reduced compared to the general population and that other health and lifestyle factors would have an impact on poorer quality of life.

## Methods

### Study population

Patients who tested positive for hepatitis C virus (HCV) by PCR (polymerase chain reaction) were approached at outpatient clinics at Memorial Sloan-Kettering Cancer Center (MSKCC), North General Hospital (NGH), and Mt. Sinai Hospital. We began the study at these institutions in June 2000, October 2001, and May 2003, respectively, following approval by the Institutional Review Boards at each site. Those eligible for the study were aged 18 years or older, English-speaking, and approved for the study by their physician. The overall response rate to the study was 66% of those approached. There were 188 patients who completed both the main questionnaire and the SF-36. Twenty-three were excluded from this analysis because they had sustained response to treatment, defined as having negative PCR for at least two measurements at least 6 months apart. Also excluded were 23 patients who completed the SF-36 while on treatment for HCV and two who completed it while being treated for cancer; they were excluded because treatment is likely to lead to short-term reduction in quality of life. This analysis is based on 140 patients.

### Collection of epidemiologic, quality of life, and clinical data

After obtaining informed consent, the medical interviewer administered a questionnaire to determine demographic characteristics, medical history, the probable route of infection, and other lifestyle factors. Information on health-related quality of life was collected by use of the SF-36. This is a validated and frequently-used instrument that includes 36 questions on quality of life, grouped into eight domains: physical functioning, general health perception, pain, social functioning, role limitations-emotional, role limitations-physical, vitality, and mental health. These eight domains can be summarized in two overall measures, the Physical Component Scale (PCS) and the Mental Component Scale (MCS). Using standardized methods [7,8], we recalibrated raw scores as required, imputed missing values for individual questionnaire items where possible, and transformed scores for each domain to a scale of 0 to 100.

We calculated summary measures for the PCS and MCS and transformed them using norm-based scoring, which results in a mean of 50 and SD of 10 in the general U.S. population [8]. Because of missing data on some of the domains, the number of patients with PCS and MCS scores was 136. Higher scores on these measures indicate better health. Information on clinical factors, such as treatment, stage, and ALT level was abstracted from medical records. Data on ALT were missing for two patients.

### Statistical methods

Data were analyzed using SAS. Normative data on the PCS and MCS summary scales have been published for the general U.S. population, based on a survey conducted in 1990 [8]. We compared mean scores of our patients to these norms, in total and for men and women separately. We used t-tests for independent samples to compare means on the eight domains and on the PCS and MCS summary scales in subgroups of our patients defined by demographic factors (e.g., gender) and health factors (e.g., presence of chronic conditions). For those variables for which statistically significant differences were found in the PCS and MCS between subgroups of patients, we used multivariate general linear models to determine which of these variables were independently related to the PCS and MCS scores. Because of our interest in the effects of cancer history, we also included this factor in these models.

## Results

### Characteristics of patients

About half of the patients in this study were men (53%) and the mean age was 53.4 years (SD 11.4, range 23 to 87). Men were younger than women (50.8 vs 56.3, p < 0.01). Eighty-six percent were from MSKCC, with most of the rest from NGH. About half were Caucasian and about one-third were African-American. While more than half had at least some college education, 20% had not graduated from high school. Forty-seven percent were currently employed. Two-thirds were physically active when interviewed, participating in physical activities such as walking, sports, aerobics, running or other activity more than once a week. Forty-eight percent had a previous diagnosis of cancer. These are mainly long-term cancer survivors, with a mean of 9.4 years since diagnosis (median 6.6 years, range 4 months to 38 years).

Diagnosis of cancer and years since diagnosis were the same in men and women. Among these patients, most had breast cancer (n = 17), followed by lymphoma (n = 14), colon cancer (n = 7), testicular cancer (n = 6), prostate cancer (n = 5) and leukemia (n = 5). Among all patients, other conditions were common: 72% had at least one chronic medical condition (heart disease, diabetes, hypertension, lung disease, thyroid disease, arthritis, rheumatoid arthritis, asthma, stroke or transitional ischemic attack, Crohn's disease, colitis, ulcers, or psoriasis); the highest prevalences were for hypertension (37%) and arthritis (22%). Forty-three percent had two or more of these conditions. Women were more likely than men to have heart disease and thyroid disease and to have two or more conditions.

One-quarter of the patients had been diagnosed with depression at some time and 15% were currently being treated for depression; more women than men ever had depression. Using responses to questions on the amount of beer, wine, and hard liquor drunk and the frequency of drinking each type of alcohol, we determined that 36% of respondents had a history of high alcohol consumption, defined as drinking an average of >2 drinks per day of beer, wine, or hard liquor for men and >1 drink per day for women [9]. Among those with heavy use of alcohol, 22% continued to drink in the last year, while among those who had stopped drinking, the mean number of years since drinking was 8.8 ± 7.4 (range, 1–30 years). The most common route of infection was IV drug use, for 40%, with 30% infected through transfusions and 30% infected through other or unknown routes. The mean number of years since using IV drugs was 19, and none claimed to have used IV drugs in the past 1.5 years. Sixteen patients (11%) had a positive test for human immunodeficiency virus (HIV) by self-report. Four percent were treated for hepatitis C before completing the SF-36; the mean number of months between completing treatment and filling out the questionnaire was 11.7 ± 10.1 (range 1–27 months). An additional 18% were treated after filling out the SF-36. ALT levels close to the time of completing the SF-36 questionnaire (mean 19.7 days, range 0–163) were available for 137 patients: the median was 56 and 20% had ALT levels of 100 or above. Among the 81 patients who had liver biopsy, 42% had stage III-IV disease, including 12% with stage IV.

### Comparison with population norms

Table 1 shows the results for the summary measures, the PCS and MCS. Compared to the norm of 50 among the general U.S. population, patients with CHC scored lower on these measures, particularly the PCS. Compared to men in the general population, men with CHC scored below the norm for men on the PCS, but were equal to the norm on the MCS. In contrast, women with CHC scored below the norms for women on both these summary scales.

**Table 1:** Mean scores (SD) on PCS and MCS for patients with CHC and general U.S. population

	**Total**		**Men**		**Women**	
	CHC patients (n = 136)	General population (n = 2474)	CHC patients (n = 72)	General population (n = 1055)	CHC patients (n = 64)	General population (n = 1412)
PCS	45.4^a ^(10.6)	50.0 (10)	47.3^b ^(10.1)	51.0 (9.4)	43.3^a ^(10.9)	49.1 (10.4)
MCS	48.2^c ^(11.1)	50.0 (10)	50.7 (9.4)	50.7 (9.6)	45.3^b ^(12.2)	49.3 (10.3)

General population data from Ware & Kosinski, 2001

^a ^p < 0.0001

^b ^p < 0.01

^c ^p < 0.05

### Domains of quality of life

Table 2 shows scores for the individual domains. The first column shows transformed results, scaled to the norm of 50; transformation accounts for differences in the structure of the scales and allows for comparison between scales. The second column shows scores before transformation, to facilitate comparison to other studies. On the transformed scores, the CHC patients in our study had the highest scores for vitality and role-emotional (i.e., the extent to which emotional problems interfere with daily activities), and the lowest for general health.

**Table 2:** Mean scores (SD) on eight domains

	Transformed to norms (n = 140)	Not transformed (n = 140)
Physical functioning	45.6 (11.3)	72.7 (27.8)
Role limitation – physical	46.3 (9.3)	66.3 (33.7)
Bodily pain	47.0 (12.4)	64.1 (29.1)
General health	44.0 (11.4)	57.1 (24.9)
Vitality	48.7 (11.4)	54.7 (24.5)
Social functioning	45.5 (12.8)	73.8 (29.7)
Role – emotional	48.0 (8.8)	78.5 (27.1)
Mental health	46.4 (12.2)	69.2 (21.5)

### Factors associated with PCS and MCS

In univariate analysis, shown in Table 3, female gender, lower physical activity, history of heavy alcohol use and history of depression led to lower scores on both the PCS and MCS. Other variables that were associated with significantly lower PCS scores on univariate analysis were having less than a high school education, not working currently, and having two or more chronic conditions. Those with a high ALT level (i.e., ALT >100) close to the time of completing the SF-36 had lower scores on the PCS that were marginally significant. We included these variables in multivariate analyses of factors affecting the PCS score. In addition, because of our interest in quality of life among cancer patients with CHC, we included history of cancer in the models.

**Table 3:** Patient characteristics and mean PCS and MCS scores (SD) according to patient characteristics

		**#**	**%**	**PCS**	**MCS**
Total		136	100	45.4 (10.6)	48.2 (11.1)
Gender	Male Female	72 64	53 47	47.3 (10.1) 43.3 (10.9)^a^	50.7 (9.4) 45.3 (12.2)^b^
Education	<12 years ≥12 years	28 108	21 79	40.8 (10.9) 46.6 (10.3)^b^	45.9 (12.0) 48.7 (10.8)
Currently employed	No Yes	74 62	54 46	42.4 (10.7) 49.0 (9.4)^c^	47.5 (11.7) 48.9 (10.3)
Physical activity^d^	≤once/week >once/week	44 92	32 68	41.8 (11.4) 47.1 (9.9)^b^	44.6 (12.3) 49.9 (10.1)^b^
History of cancer	Yes	65	48	43.8 (10.1)	47.7 (10.4)
	No	71	52	46.9 (11.0)	48.6 (11.7)
Number of chronic conditions^e^	<2 ≥2	76 60	56 44	48.8 (9.9) 41.1 (10.0)^c^	49.7 (8.8) 46.2 (13.3)
History of depression	Yes	36	26	40.3 (10.3)	41.9 (10.6)
	No	100	74	47.2 (10.2)^c^	50.4 (10.4)^c^
ALT	≥100	26	20	42.0 (10.1)	49.9 (9.4)
	<100	107	80	46.4 (10.6)	47.8 (11.3)
History of heavy alcohol use^f^	Yes	49	36	42.6 (11.1)	45.1 (11.7)
	No	87	64	47.0 (10.1)^a^	49.9 (10.4)^a^

^a ^p < 0.05 ^b ^p < 0.01 ^c ^p < .001. Numbers shown are for total sample, although actual number varies slightly for individual scales.

^d ^patients with high levels of physical activity participated in activities such as walking, jogging, active sports, aerobic exercises, and heavy physical activity at work more than once a week

^e ^includes heart disease, diabetes, hypertension, lung disease, thyroid disease, arthritis, asthma, stroke or transitional ischemic attack, Crohn's disease, colitis, ulcers, and psoriasis

^f ^>2 drinks of beer, wine, or liquor per day, on average, for men, and >1 drink per day for women.

Results of multivariate models for the PCS incorporating these variables are shown in Table 4. History of cancer and having two or more other conditions were strongly associated with the PCS, and high ALT level, having fewer years of education, and low physical activity also had a significant effect on the PCS score. Although gender had an important effect in univariate analysis, it was strongly related to education and depression, with women having less education and higher prevalence of depression; with all these variables in the model, only education had an independent effect. Current employment and alcohol use were not significant in multivariate analysis. The variables in the model explained 39% of the variation in the PCS. Results of multivariate analysis differed by gender: among men, only a history of cancer was associated with the PCS, while among women, only chronic conditions affected the score. The variables included in the models explained more of the variance for women than for men.

**Table 4:** F values for factors associated with PCS in multivariate analysis in total and by gender

	**Total (n = 133)**	**Men (n = 71)**	**Women (n = 62)**
Female gender	2.27	NA	NA
<12 years education	5.68^a^	3.36	2.67
Not currently employed	2.90	0.40	1.01
Low physical activity	5.50^a^	3.12	0.93
History of cancer	10.13^b^	9.98^b^	1.15
≥2 chronic conditions	15.00^c^	3.14	12.99^c^
History of depression	2.72	0.39	3.97
ALT ≥100	8.26^b^	3.97	3.65
History of heavy alcohol use	1.65	0.15	2.23
r^2^	.39	.31	.51

NA – not applicable

^a ^p < 0.05 ^b ^p < 0.01 ^c^p < 0.001

In univariate analysis (Table 3), the factors associated with the MCS were depression, level of physical activity, gender, and alcohol consumption. These factors and cancer history were included in multivariate models. Results, shown in Table 5, indicate that each of these factors, except history of cancer, was independently associated with MCS, explaining 23% of the variance. Among men, only physical activity was significant, and among women, depression and alcohol use were significant.

**Table 5:** F values for factors associated with MCS in multivariate analysis in total and by gender

	**Total (n = 135)**	**Men (n = 72)**	**Women (n = 63)**
Female gender	5.92^a^	NA	NA
Low physical activity	6.88^b^	8.43^b^	0.48
History of cancer	1.74	0.91	0.29
Depression	9.38^b^	3.14	4.77^a^
History of high alcohol use	5.59^a^	1.43	4.06^a^
r^2^	.21	.16	.21

NA – not applicable

^a ^p < 0.05 ^b ^p < 0.01

We also investigated several other factors for their association with the PCS and MCS, in both univariate and multivariate analyses. Variables not related to these quality of life measures included: age, race, marital status, hospital, HIV infection, route of transmission, treatment for CHC, stage of fibrosis, and measures of social support (attendance at religious services, attendance at meetings of community groups, and number of friends).

## Discussion

As we hypothesized, quality of life was reduced in this population and was related to other physical and lifestyle conditions in these study participants. Our extensive epidemiologic information allowed us to study a number of factors that have not been addressed in other studies and provides an understanding of how these factors affect quality of life in patients with HCV.

Six other studies [10-15] have analyzed health-related quality of life in patients with CHC in clinic settings. Untransformed scores on the eight domains among our patients were generally similar to those reported in these studies, although on several domains our scores tended to be somewhat higher. Hussain et al. [11] reported PCS and MCS summary scores and results according to several patient characteristics. Comparing our results to those, patients in the present study had similar scores on the PCS (45 vs 44) and significantly higher scores on the MCS (48 vs 44, p < 0.01).

Differences in patient characteristics make comparisons somewhat uncertain; our study included higher proportions of women and a lower proportion who were employed, but a higher proportion with at least a high school education. Consistent with our results from univariate analysis, Hussain et al. [11] reported that women, those with less education, and those with comorbid illnesses had lower scores on the PCS. The two studies were also consistent in reporting no association with age or drug use. Consistent results were also reported by Fontana et al. [12], who found that painful comorbid conditions, such as arthritis or migraine headaches, and current depression were associated with poorer quality of life in a study in patients for whom previous interferon therapy had failed. Two other studies considered the association between ALT measures and quality of life [10,15], and did not find an association, as we did. Earlier studies that investigated patient characteristics did not use multivariate methods to investigate the independence of the factors studied. In the present study, multivariate analysis indicated that gender per se was not significantly associated with PCS scores when related variables, depression and education, were included.

The inclusion of many patients with cancer in our setting adds further information on the effect of chronic conditions on quality of life. Overall, we found reduced quality of life on physical measures for cancer survivors compared to other CHC patients, especially in men, and no difference on measures reflecting mental health. The literature on quality of life in cancer survivors indicates that quality of life may be reduced in long-term survivors [16-20], although a number of studies have found quality of life to be similar to population norms or control groups [16,21-24]. These studies were conducted in patients with different cancers and used different instruments to measure quality of life, making generalizations difficult.

To our knowledge, the influence of physical activity has not been studied previously in patients with CHC. Although it is not possible to separate the direction of the association between physical activity and quality of life (that is, people with better quality of life in general may be more able to participate in physical activity, or those who participate may improve their quality of life), the finding does raise the question of whether an intervention aimed at increasing activity might improve quality of life in the CHC population. A small study including both dietary and physical activity interventions in overweight individuals with CHC (most with HCV) reported improvement in quality of life, particularly in those who maintained weight loss, although it was not possible to evaluate the effects of diet and exercise separately [25]. A large proportion of our patients reported being physically active, probably because they are in an urban area where walking is common.

Strengths of this study are the focus on a diverse population of patients seen in different clinics in a major metropolitan area, including a cancer hospital, the availability of extensive epidemiologic data and clinical data that allowed us to investigate the effect of other factors on quality of life, and the use of multivariate analysis to examine influences on quality of life. In contrast to several other studies [1,2,4,5,12-14], we used only the SF-36 to measure quality of life because of the extensive amount of other information we were collecting; therefore, we did not have the opportunity to investigate aspects of quality of life that are more specific to hepatitis C.

Another disadvantage of this study is the relatively low proportion of those approached who agreed to take part in the study (66%); in addition, there were 73 patients who completed the main questionnaire but not the SF-36. To some extent, the level of participation reflects the characteristics of this population, who often have other medical and social problems that make joining such studies difficult. A similar response was obtained by Hussain et al. [11], although a smaller clinical study [10] reported nearly complete participation. It seems possible that those who did not take part have poorer quality of life than those who did, which would imply that quality of life in all patients with CHC is reduced even further than that reported here and in similar studies.

## Conclusion

These results support those of other studies finding that patients with CHC have considerable impairment of quality of life. Our analysis extends these findings to show that other factors strongly influence quality of life in these patients, and that all patients with CHC are not equally at risk of reduced quality of life. In our population, quality of life on the PCS scale was affected by medical factors such as history of cancer, presence of other chronic conditions, and ALT, as well as by social and lifestyle factors such as education and physical activity. On the MCS scale, quality of life was affected by history of cancer and depression, as well as by gender, alcohol use, and lack of physical activity. In addition, women and men differed in how these factors influenced the PCS and MCS scores. Understanding the degree of reduction of quality of life and other factors that are associated with this reduction should help clinicians deal more effectively with this population.

## Authors' contributions

SHO designed the study, supervised data collection, performed statistical analysis, and wrote the initial manuscript. SI, JS, OE, TM, and CT identified and interviewed eligible patients and abstracted clinical data from medical records. TM, SS, MS, MG-G, and RCK contributed to the development of the study protocol and the instruments used and helped to draft the manuscript. All authors read and approved the final manuscript.
